# Clinicopathological and Genetic Features in Superficial Nonampullary Duodenal Epithelial Tumors

**DOI:** 10.1155/grp/1063863

**Published:** 2025-05-27

**Authors:** Atsushi Sawada, Kingo Hirasawa, Makoto Sugimori, Yuichiro Ozeki, Ryosuke Ikeda, Masafumi Nishio, Takehide Fukuchi, Ryosuke Kobayashi, Hiroaki Kaneko, Chiko Sato, Yoshiaki Inayama, Chikara Kunisaki, Shin Maeda

**Affiliations:** ^1^Division of Endoscopy, Yokohama City University Medical Center, Yokohama, Kanagawa, Japan; ^2^Department of Cancer Genome Medicine, Yokohama City University Medical Center, Yokohama, Kanagawa, Japan; ^3^Department of Gastroenterology, Yokohama City University Graduate School of Medicine, Yokohama, Kanagawa, Japan; ^4^Department of Diagnostic Pathology, Yokohama City University Medical Center, Yokohama, Kanagawa, Japan; ^5^Department of Surgery, Yokohama City University Medical Center, Yokohama, Kanagawa, Japan

## Abstract

**Background and Aim:** Superficial nonampullary duodenal epithelial tumors (SNADETs) that are pathologically classified as gastric-type might manifest a more aggressive behavior than the intestinal type. However, the details of their histologic and genetic features remain unclear because of their rarity. This study was aimed at identifying clinicopathological findings and early genomic events in gastric-type SNADETs treated with endoscopic resection.

**Methods:** We retrospectively analyzed 204 patients with SNADETs between January 2011 and September 2020. Immunohistochemical analysis for *β*-catenin and targeted exome sequence analysis of 50 cancer-related genes using next-generation sequencing were performed for the representative cases.

**Results:** Among the 204 SNADETs cases, only nine (4.4%) were gastric type; the remaining 195 cases were intestinal type. Among the gastric-type tumors, seven were adenomas and two were adenocarcinomas, whereas only three of the 195 intestinal-type tumors were adenocarcinomas. Nuclear expression of *β*-catenin was observed in three of the nine (33%) gastric-type tumors and in eight of the 10 (80%) intestinal-type tumors. The most prevalent abnormality among the 50 genes tested in gastric-type tumors was *GNAS* mutation (89%), whereas that in intestinal-type tumors was *APC* mutation (67%). All gastric-type adenocarcinomas had *GNAS* mutations as well as adenomas, while *APC* mutations were absent in intestinal-type adenocarcinomas and present in most adenomas.

**Conclusions:** *GNAS* mutations are more common in gastric-type SNADETs than in the intestinal type. As *GNAS* mutations are continuously present from adenoma to adenocarcinoma, resection at the adenoma stage is desirable.

## 1. Introduction

Superficial nonampullary duodenal epithelial tumors (SNADETs) are rare, but opportunities to detect them have increased with recent advances in endoscopic imaging [1, 2]. Less-invasive treatments for SNADETs using endoscopic resection (ER) significantly benefit patients with nonampullary duodenal adenomas because these adenomas are precancerous lesions according to the updated World Health Organization (WHO) classification [3, 4]. However, several previous studies have reported that duodenal ER, particularly endoscopic submucosal dissection, is associated with a high risk of adverse events such as perforation and bleeding [5–7]. Based on these findings, the analysis of early molecular events in SNADETs is becoming increasingly important for defining treatment indications in clinical practice.

Nonampullary duodenal submucosal invasive adenocarcinomas are rare, and their clinicopathological characteristics have not been investigated in detail [8, 9]. According to recent studies, almost all nonampullary duodenal submucosal invasive adenocarcinomas express gastric phenotypic markers (MUC5AC and/or MUC6) [10]. Because the duodenal mucosa consists of villi, crypts, and Brunner glands, SNADETs may exhibit an intestinal or gastric phenotype [11, 12]. Previous studies have demonstrated that SNADETs differ between gastric and intestinal phenotypes in terms of clinical features and pathogenesis [13–19]. Although gastric-type adenomas are extremely rare compared to intestinal-type adenomas, the gastric phenotype of duodenal adenocarcinoma shows a more aggressive biological behavior than the intestinal phenotype [8, 10, 13, 15, 16, 20]. Additionally, recent studies have suggested that the mutation status of SNADETs differs between intestinal and gastric phenotypes [21, 22]. However, only a few studies have investigated the early mechanisms underlying duodenal carcinogenesis because of the extremely low prevalence of this phenotype. We aimed to elucidate the early molecular events in gastric-type SNADETs. To this end, we performed immunohistochemical assessments to explore the role of the WNT/*β*-catenin signaling pathway and DNA mismatch repair protein (MMR) abnormalities. We also evaluated genomic features using next-generation sequencing (NGS).

## 2. Methods

### 2.1. Patients

Two hundred four patients with SNADETs who presented to Yokohama City University Medical Center between January 2011 and September 2020 were eligible for this study. All lesions were obtained using ER. We excluded patients with polyposis syndromes, such as familial adenomatous polyposis, and those with inflammatory bowel diseases, such as Crohn's disease.

All samples included in this study were evaluated in accordance with the principles of the Declaration of Helsinki. The study and its protocol were approved by the ethical review board of our hospital (Yokohama City University Certified Institutional Review Board; A180524005, D1602024). All patients were informed of the risks and benefits of the treatments before they underwent the procedures. Informed consent was obtained from all patients included in the study.

### 2.2. Clinicopathological Definition

The following baseline characteristics were recorded: patient age, sex, tumor diameter, tumor location, macroscopic type, morphologic phenotype, tumor diameter, and histological diagnosis according to the WHO classification [3, 23].

### 2.3. Histological Classification

Resected specimens were fixed in 10%–20% formalin, serially sectioned at 2-mm intervals, and embedded in paraffin. From each paraffin block, 4-*μ*m sections were obtained and stained with hematoxylin and eosin (H&E) and microscopically examined for histopathological findings by two or more expert pathologists. All specimens were classified according to the WHO criteria by the pathologists who were blinded to the results of immunochemical and genetic analyses [3, 23]. The results of the histological and immunohistochemical evaluations were determined via consensus between at least two pathologists.

All noninvasive neoplastic lesions were classified as adenomas, which were further categorized as low- or high-grade and gastric- or intestinal-type, according to WHO criteria and previous studies [3, 20, 21, 23, 24]. Adenomas with high-grade dysplasia covering more than 10% of the area were classified as high-grade lesions. High-grade dysplasia is characterized by architectural complexity and/or significant cytologic atypia [21]. Gastric-type adenomas include pyloric gland adenomas (PGAs) and foveolar-type adenomas (FAs) [24]. If gastric-type adenomas did not fulfill the diagnostic criteria for PGAs and FAs, they were defined as gastric, not otherwise specified (NOS) type. All invasive neoplastic lesions were classified as adenocarcinomas, which were classified as gastric and intestinal types based on morphology. When both morphologic phenotypes were observed in a single tumor, the tumor was evaluated according to its predominant component.

### 2.4. Immunohistochemical Analysis

Immunohistochemical staining was performed on representative sections obtained from the tumor with the largest diameter. The primary antibodies used in this study are listed in Supporting Information 1: Table S1. The mucin phenotype was evaluated using MUC2, MUC5AC, MUC6, CD10, and CDX2. SNADETs are classified into two types according to their mucin phenotype: gastric and intestinal. Intestinal-type tumors were identified using CD10, CDX2, and MUC2 staining. Gastric-type tumors were identified by staining for MUC5AC and MUC6. When both phenotypes were observed in a single tumor, the tumor was evaluated based on its predominant component.

**Figure 1:**
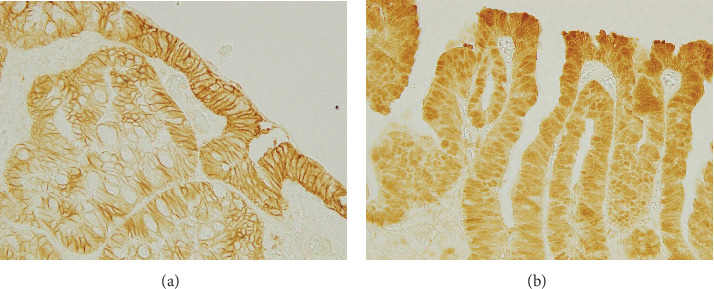
*β*-Catenin expression in superficial nonampullary duodenal epithelial tumors (SNADETs). (a) Representative case of gastric-type tumors showing negative nuclear expression of *β*-catenin expression. (b) Representative case of intestinal-type tumors showing positive nuclear expression of *β*-catenin expression.

Immunohistochemical studies of *β*-catenin expression were conducted according to a previous report [25]. Abnormal nuclear staining for *β*-catenin greater than 5% was considered positive [25] (Figure 1). Immunohistochemical studies of MMR proteins MLH1, PMS2, MSH2, and MSH6 were conducted according to a previous report [26]. Adjacent non-neoplastic tissue was used as an internal control for each slide, and staining was interpreted only when normal tissues adjacent to the neoplasm showed distinct nuclear labeling. The presence of protein expression in the tumor nuclei was evaluated. Neoplasms with diffuse loss of MMR proteins are regarded as deficient MMR expression.

### 2.5. Genetic Analysis

Formalin-fixed paraffin-embedded (FFPE) specimens were cut into 4- and 15-*μ*m thick sections for the detection of tumor tissue and laser microdissection (LMD), respectively. LMD was conducted to obtain as many cancer cells as possible. The quantity and quality of the extracted DNA were assessed using a NanoDrop spectrophotometer (Thermo Fisher Scientific). A ready-made gene panel (Ion AmpliSeqCancer Hotspot Panel v.2, Thermo Fisher Scientific, Waltham, MA, United States) was used to amplify 50 cancer-related target genes. A total of 2790 hotspot mutations in 50 cancer-related genes have been reported in the Catalogue of Somatic Mutations in Cancer (COSMIC; hotspot mutations) Database 8. The analyzed genes were as follows: *ABL1*, *AKT1*, *ALK*, *APC*, *ATM*, *BRAF*, *CDH1*, *CDKN2A*, *CSF1R*, *CTNNB1*, *EGFR*, *ERBB2*, *ERBB4*, *EZH2*, *FBXW7*, *FGFR1*, *FGFR2*, *FGFR3*, *FLT3*, *GNA11*, *GNAS*, *GNAQ*, *HNF1A*, *HRAS*, *IDH1*, *JAK2*, *JAK3*, *IDH2*, *KDR*, *KIT*, *KRAS*, *MET*, *MLH1*, *MPL*, *NOTCH1*, *NPM1*, *NRAS*, *PDGFRA*, *PIK3CA*, *PTEN*, *PTPN11*, *RB1*, *RET*, *SMAD4*, *SMARCB1*, *SMO*, *SRC*, *STK11*, *TP53*, and *VHL*. Data analysis was performed using Ion Reporter software: allele frequency (AF) > 10% and coverage (Cov.) results > 200 were defined as positive [27].

### 2.6. Statistical Analysis

Statistical analysis was performed with JMP 15 (SAS Institute Inc., Cary, NC, United States). The proportions of categorical variables were compared using the two-sided Fisher's exact test or chi-squared test. Continuous variables were compared using the Wilcoxon–Mann–Whitney test. A *p* value < 0.05 was considered significant.

## 3. Results

### 3.1. Clinicopathological Features

The characteristics of patients and lesions are summarized in Table 1. Regarding the histological diagnosis, 199 (97%) and five (3%) SNADETs were diagnosed as adenomas and adenocarcinomas, respectively. The five adenocarcinomas were confined to the submucosa. Details of the clinical outcomes of these five patients are provided in Supporting Information 2: Table S2. No significant differences were observed in sex, age, tumor diameter, and tumor location between adenomas and adenocarcinomas. The macroscopic appearance of the protruding phenotype significantly correlated with that of an adenocarcinoma (*p* < 0.001).

### 3.2. Results of Immunohistochemical Studies

**Figure 2:**
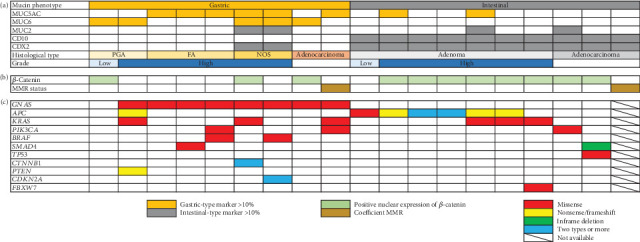
Molecular and clinicopathological features of superficial nonampullary duodenal epithelial tumors (SNADETs). (a) Mucin phenotypes, histological types, and degrees of dysplasia. (b) *β*-Catenin expression status and mismatch repair protein (MMR) status. (c) The summarized results of targeted sequencing of cancer-related genes in tumors of the indicated histological types.

Among the 204 SNADETs cases, only nine (4.3%) were gastric type, and the remaining 195 cases were intestinal type as determined by H&E histology. Seven cases were adenoma and two gastric-type tumors were adenocarcinomas, whereas only three of the 195 intestinal-type tumors were adenocarcinomas. The proportion of the gastric-type phenotype was significantly higher in adenocarcinomas than in adenomas (*p* < 0.001). We examined the elements of major pathways, including the Wnt/*β*-catenin signaling pathway and the DNA repair mechanism, to investigate early molecular events in SNADETs. Immunochemical analysis was performed on the 19 lesions: nine gastric-type tumors (two adenocarcinomas and seven adenomas) and 10 intestinal-type tumors (three adenocarcinomas and seven adenomas, which were randomly selected from the 192 intestinal-type adenomas as controls). The immunohistochemical features of the mucin phenotype were investigated in these 19 lesions. The result of the mucin phenotype was consistent with that of the morphologic phenotype (Figure 2).

The characteristics of the 19 patients and their lesions are summarized in Table 2. Gastric-type tumors were histologically divided into low-grade adenomas (*n* = 1), high-grade adenomas (*n* = 6), and adenocarcinomas (*n* = 2). Additionally, seven adenomas were subdivided into PGAs (*n* = 2), FAs (*n* = 3), and NOS-type adenomas (*n* = 2). One adenocarcinoma sample consisted of MUC6-positive atypical cells growing in a glandular pattern. The other adenocarcinoma consisted of MUC5AC-positive atypical cells growing in a glandular pattern. All gastric-type tumors were located on the oral side of the ampulla of Vater. Macroscopically, most gastric-type tumors appeared protruding. In contrast, intestinal-type tumors were histologically classified as low-grade adenomas (*n* = 1), high-grade adenomas (*n* = 6), and adenocarcinomas (*n* = 3). Macroscopically, most intestinal-type tumors appeared flat.

#### 3.2.1. *β*-Catenin Immunohistochemistry

Gastric-type tumors showing positive nuclear expression of *β*-catenin were identified in three of the nine cases (33%) (Figure 2). Specifically, they were identified as PGAs, adenocarcinomas consisting of diffuse MUC6-positive atypical cells, and NOS-type adenomas. In contrast, gastric-type tumors showing negative nuclear expression of *β*-catenin were identified as FAs, and the adenocarcinomas consisted of MUC5AC-positive atypical cells and NOS-type adenomas. Furthermore, intestinal-type tumors showing abnormal nuclear expression of *β*-catenin were identified in eight of the 10 (80%) patients.

#### 3.2.2. MMR Immunohistochemistry

**Figure 3:**
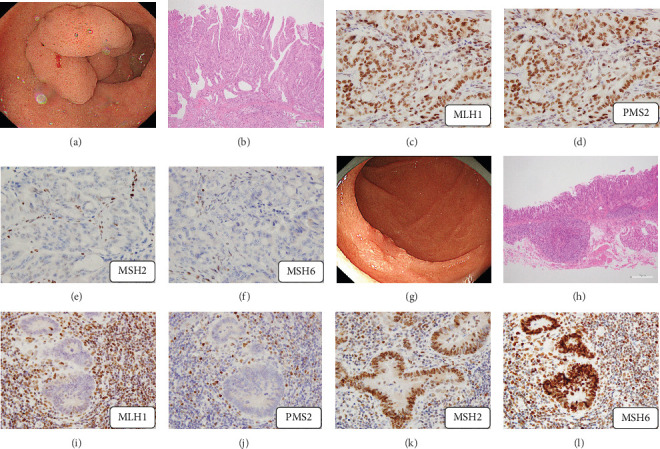
Immunohistochemical staining of deficient mismatch repair protein (MMR). (a–f) Endoscopic and histological images of a case of gastric-type adenocarcinoma showing the loss of MSH2 and MSH6 expression. (g–l) Endoscopic and histologic images of a case of intestinal-type adenocarcinoma showing the loss of MLH1 and PMS2 expression.

Of the 19 SNADETs, two showed deficient MMR; one was a gastric-type adenocarcinoma that showed loss of MSH2 and MSH6, and the other was an intestinal-type adenocarcinoma that showed loss of MLH1 and PMS2 (Figure 3). A review of clinical records revealed that intestinal-type adenocarcinoma occurs in patients with genetically confirmed Lynch syndrome.

### 3.3. Mutation Profiles

We performed cancer-related gene target sequencing analysis to investigate early genetic events in SNADETs. NGS analysis was performed on the same lesions as in the immunochemical analysis. Nineteen libraries were sequenced using NGS. One library could not be analyzed because of sample errors. Ultimately, 18 libraries were analyzed using NGS. The distribution of somatic mutations is summarized in Figure 2. The most prevalent abnormality among the 50 genes tested in gastric-type tumors was a *GNAS* mutation, which was found in eight of nine (89%) patients, followed by *KRAS* mutations in three of nine (33%) patients, *PIK3CA* mutations in two of nine (22%) patients, and *BRAF* mutations in two of nine (22%) patients. In contrast, the most prevalent abnormality in intestinal-type tumors was *APC* mutation, which was found in seven of nine (78%) patients, followed by *KRAS* mutations in three of nine (33%) patients. All gastric-type adenocarcinomas had *GNAS* mutations. In contrast, all intestinal-type adenocarcinomas did not have *APC* mutations; instead, *TP53*, *SMAD4*, *PIK3CA*, and other mutations were found.

## 4. Discussion

In this study, we examined the expression of *β*-catenin and MMR using immunohistochemical staining and cancer-related gene target sequencing to elucidate early molecular events in SNADETs. We found a high frequency of abnormalities in the WNT/*β*-catenin signaling pathway in most intestinal-type tumors and some gastric-type tumors. Regardless of the mucin phenotype, all duodenal adenomas were proficient in MMR. Two adenocarcinomas (one each from gastric and intestinal phenotypes) were deficient in MMR. Additionally, we observed a high frequency of *GNAS* mutations in gastric-type adenomas and *APC* mutations in intestinal-type adenomas, with a tendency towards mutual exclusivity. Although the number of cases was limited, *APC* mutations were not detected in intestinal-type adenocarcinomas in this study. In contrast, *GNAS* mutations have been detected in all gastric-type adenocarcinomas. Additionally, the carcinogenesis of SNADETs may involve deficient MMR and additional mutations, such as *TP53*, *PIK3CA*, *SMAD4*, and *KRAS*.

Recent studies have suggested that the WNT/*β*-catenin signaling pathway is involved in the development of nonampullary adenomas with the intestinal phenotype [17, 22, 25]. Similarly, we observed a high frequency of intranuclear expression of *β*-catenin and *APC* mutations in intestinal-type adenomas. However, the pathogenesis of gastric-type duodenal neoplasms remains poorly understood owing to their rarity. In this study, intranuclear *β*-catenin expression was identified in 33% of gastric-type tumors. Similar to recent reports, our data suggest that the WNT/*β*-catenin signaling pathway is less involved in the development of gastric-type tumors [17, 22]. Although the recent WHO classification only refers to pyloric gland differentiation in gastric phenotype neoplasms of the duodenum, the differentiation of gastric-type SNADETs varies as with gastric neoplasms [3]. In the present study, intranuclear *β*-catenin expression suggested the presence of gastric-type tumors with pyloric gland differentiation, while the absence of *β*-catenin intranuclear expression indicated gastric-type tumors with foveolar epithelial differentiation. These findings suggest that signaling pathways that directly promote gastric-type tumor progression differ between pyloric gland and foveolar epithelial differentiation.

Few immunohistochemical studies have used antibodies against the four major MMR proteins in SNADETs [28]. In the present study, no samples with loss of MMR proteins were detected in duodenal adenomas. Similarly, recent studies have suggested that deficient MMR is rare in duodenal adenomas [21, 22, 28]. A systematic review found that microsatellite instability (MSI) in colorectal adenomas was present in 2.8% of patients with no history of Lynch syndrome [29]. Considering these findings, it is clear that the detection of deficient MMR/MSI in duodenal adenomas is difficult in clinical practice, similar to that in colorectal adenomas. In contrast, we detected two samples with loss of MMR among the five adenocarcinomas; the proteins lost were MLH1 and PMS2 in the intestinal-type case, and MSH2 and MSH6 in the gastric-type case (Figure 3). Similarly, previous studies have demonstrated that small bowel carcinomas, particularly duodenal carcinomas, have a higher incidence of MSI-high than other gastrointestinal carcinomas [30, 31]. Solid cancers showing MSI-high tumors are caused by somatic mutations or epigenetic changes. The diagnosis of Lynch syndrome is often missed in clinical practice owing to a lack of knowledge of the early stages of cancers. A large-scale multicenter analysis of ER for SNADETs revealed that 1.4% of patients had carcinoma with submucosal invasion [1]. Our results support the conclusion that the final pathological diagnosis after ER for SNADETs is adenocarcinoma, and immunohistochemical examination of MMR should be conducted for the screening of Lynch syndrome.

Recent studies have demonstrated that *APC* mutations are less common in duodenal carcinomas, suggesting that the adenoma–carcinoma sequence is limited to intestinal-type duodenal carcinomas [21, 22, 30, 32, 33]. Although the number of cases was limited, our genetic analyses of duodenal adenomas/carcinomas showed similar trends. In contrast to the genetic results, our study showed nuclear *β*-catenin expression in 60% of adenocarcinomas. Our results suggest that upregulation of the WNT/*β*-catenin pathway due to various causes other than *APC* mutations is an important factor in the carcinogenesis of SNADETs.

In the present study, gastric-type SNADETs were more complicated than intestinal-type SNADETs in terms of the histological subtype of the mucin phenotype. A recent study also showed that the histological subtype of the gastric phenotype neoplasm varied and was similar to that of gastric phenotype neoplasms of the stomach [24]. We classified gastric phenotype tumors of the duodenum into three subtypes: pyloric gland, foveolar, and NOS, all of which had common *GNAS* mutations. *GNAS* mutations are rare in adenocarcinomas arising in the gastrointestinal tract and are considered histologically specific genetic mutations [34–36]. Despite the rarity of *GNAS* mutations in adenocarcinomas, both gastric-type duodenal adenomas and adenocarcinomas frequently harbor *GNAS* mutations. Regarding morphologic phenotypes, the gastric type was rare among duodenal adenomas (4%, 7/199), while it was common among duodenal adenocarcinomas (40%, 2/5). In addition, according to our preliminary observations, among the five advanced nonampullary adenocarcinomas treated via surgical resection during the same period as the current study period, four (80%) were gastric type, as determined by H&E histology. These observations imply that ER may be recommended for gastric-type adenomas, similar to colonic adenomas. However, *GNAS* mutations are also common in gastric foveolar metaplasia and ectopic gastric mucosa, and the most effective treatment for the specific stage of the gastric metaplasia–adenoma–carcinoma sequence remains controversial. Additionally, previous studies have reported that the frequency of *GNAS* mutations is at most 10% in duodenal adenocarcinomas, suggesting that most duodenal adenocarcinomas are not derived from gastric-type duodenal adenomas [21, 30]. However, in contrast to our data, approximately 30% of duodenal adenocarcinomas and most duodenal adenomas have *APC* mutations [30]. Thus, ER of intestinal-type adenomas is recommended rather than their follow-up. However, the previous report only targeted sporadic lesions and found that less than 10% of duodenal adenocarcinomas had *APC* mutations [21]. Further large-scale studies are needed to determine the indications for endoscopic treatment of sporadic intestinal-type adenomas. Delving deeper into the indication for endoscopic treatment, it is necessary to discuss the presence or absence of preoperative biopsy. Several studies have reported that preoperative biopsy is the source of submucosal fibrosis, which results in difficulty in performing ER for SNADETs [37]. Given the relatively low accuracy of duodenal biopsy sampling, it is crucial to emphasize preoperative endoscopic diagnosis when assessing the indication for ER. Recent research suggests that the mucin phenotype of SNADETs can be identified through preoperative endoscopic examination [38]. Based on our research and current findings, it may be preferable to proceed with endoscopic treatment rather than biopsy when diagnosing gastric-type SNADETs during preoperative endoscopy [37, 38].

This study had several limitations. First, the sample size was small owing to the rarity of gastric-type tumors. Similarly, evaluating tumors with mixed phenotypes for submucosal invasive cancer was challenging for the same reasons. The small number of cases is an inherent limitation of our study. Additional larger multicenter studies investigating the molecular events involved in gastric-type SNADETs are required. Second, the immunohistochemical staining and genetic findings of most duodenal adenomas were not examined. Therefore, the generalizability of our finding, that is, the relationship between higher malignancy in the gastric type and the observation that GNAS mutations are more frequently expressed in the gastric type, suggesting a lesser involvement of the Wnt/*β*-catenin pathway, is limited. Third, we investigated mutations in only 50 of these genes. Therefore, we could not analyze other gene alterations in SNADETs. Fourth, analysis of the corresponding normal samples to validate somatic mutations was not performed. Despite these limitations, the present study provides unique insights into the foundation for studies of the molecular mechanisms underlying duodenal carcinogenesis, which may eventually promote the decision of the indication for treatment of SNADETs.

## 5. Conclusions

The results of our analysis demonstrate that gastric-type SNADETs are extremely rare and frequently harbor *GNAS* mutations in both adenomas and adenocarcinomas. Therefore, ER is required for precancerous gastric-type adenomas. Further studies are required to determine the priority of SNADETs treatment based on the mucin phenotype.

## Figures and Tables

**Table 1:** Patient and lesion characteristics between nonampullary duodenal adenoma and adenocarcinoma.

	**Adenoma (** **n** = 199**)**	**Adenocarcinoma (** **n** = 5**)**	*p * ** value**
Sex (male/female) (%)	123/76 (62/38)	2/3 (40/60)	0.93
Age, median (range, years)	68 (34–91)	70 (51–84)	0.32
Macroscopic appearance (protruding/flat) (%)	32/167 (16/84)	4/1 (80/20)	< 0.001
Tumor diameter, median (range, mm)	10 (3–43)	10 (8–31)	0.57
Tumor location (oral side of the AV/anal side of the AV) (%)	97/102 (49/51)	3/2 (60/40)	0.61
Morphologic phenotype (gastric/intestinal) (%)	7/192 (4/96)	2/3 (40/60)	< 0.001

Abbreviation: AV, ampulla of Vater.

**Table 2:** Clinicopathological characteristics of all 19 patients.

	**Gastric type (** **n** = 9**)**	**Intestinal type (** **n** = 10**)**
Sex (male/female)	6/3	7/3
Age, median (range, years)	68 (34–91)	71 (51–84)
Tumor diameter, median (range, mm)	11 (8–31)	12 (8–28)
Tumor location (oral side of the AV/anal side of the AV)	9/0	5/5
Macroscopic type (protruding/flat)	8/1	2/8
Histology (adenoma/adenocarcinoma)	7/2	7/3

Abbreviation: AV, ampulla of Vater.

## Data Availability

The data used to support the findings of this study are available from the corresponding author upon request.

## Supporting Information

Supporting Information 1 Table S1: Detailed information on the antibodies used for immunohistochemistry, including clone names, sources, and dilution ratios.

Supporting Information 2 Table S2: Clinicopathologic characteristics of five patients with submucosal invasive carcinomas, including tumor size, location, depth of invasion, lymphovascular invasion, and clinical outcomes.
