# Supplementary material for: Clinicopathological and Genetic Features in Superficial Nonampullary Duodenal Epithelial Tumors
Source: Gastroenterol Res Pract. 2025 May 27;2025:1063863. doi: 10.1155/grp/1063863 (PMC12133369; doi:10.1155/grp/1063863)
Supplement: Supporting Information 1 — Table S1: Detailed information on the antibodies used for immunohistochemistry, including clone names, sources, and dilution ratios. [file 1063863.f1.docx]

| **Supplemental Table 1.** Antibodies used for immunohistochemistry | | | |
| --- | --- | --- | --- |
| Maker | Clone | Manufacturer | Dilution |
| β-catenin | 14 | Cell Marque | 1:50 |
| MLH1 | M1 | Ventana | None |
| MSH2 | G219-1129 | Ventana | None |
| MSH6 | SP93 | Ventana | None |
| PMS2 | A16-4 | Ventana | None |
| MUC2 | Ccp58 | Leica | None |
| MUC6 | CLH5 | Leica | None |
| MUC5AC | CLH2 | Leica | None |
| CD10 | SP67 | Roche | None |
| CDX2 | DAKO-CDX2 | Dako | None |
