# Supplementary material for: Clinicopathological and Genetic Features in Superficial Nonampullary Duodenal Epithelial Tumors
Source: Gastroenterol Res Pract. 2025 May 27;2025:1063863. doi: 10.1155/grp/1063863 (PMC12133369; doi:10.1155/grp/1063863)
Supplement: Supporting Information 2 — Table S2: Clinicopathologic characteristics of five patients with submucosal invasive carcinomas, including tumor size, location, depth of invasion, lymphovascular invasion, and clinical outcomes. [file 1063863.f2.docx]

| **Supplemental Table 2.** Clinicopathologic characteristics of five patients with submucosal invasive carcinomas. | | | | | | | | | |  |
| --- | --- | --- | --- | --- | --- | --- | --- | --- | --- | --- |
| Case | Age | Sex | Location | Morphologic  phenotype | Treatment | Resection  margin | Long-term  outcome | DFS,  days | OS,  days |  |
|  |  |  |  |  |  |  |  |  |  |  |
|  |  |  |  |  |  |  |  |  |  |  |
| 1 | 82 | M | Oral side of the AV | Intestinal | CEMR | Lateral Positive | Died of duodenal cancer | 121 | 268 |  |
| 2 | 51 | F | Anal side of the AV | Intestinal | ESD | Negative | Metachronous recurrence  of ileum segment cancer | 1708 | 2268 |  |
| 3 | 70 | F | Oral side of the AV | Gastric | ESD followed  by surgery | Negative | Died of duodenal cancer | 440 | 873 |  |
| 4 | 68 | M | Anal side of the AV | Gastric | UEMR | Negative | Died of lung cancer | 49 | 233 |  |
| 5 | 84 | M | Oral side of the AV | Gastric | UEMR | Negative | Alive without recurrence | 788 | 788 |  |
| DFS: disease-free survival; OS: overall survival; CEMR: conventional endoscopic mucosal resection; UEMR: underwater endoscopic mucosal resection; ESD:endoscopic submucosal dissection; AV: the ampulla of Vater | | | | | | | | | |  |
|  |  |  |  |  |  |  |  |  |  |  |
